# Radiomics as a non-invasive adjunct to Chest CT in distinguishing benign and malignant lung nodules

**DOI:** 10.1038/s41598-023-46391-7

**Published:** 2023-11-04

**Authors:** Minmini Selvam, Anupama Chandrasekharan, Abjasree Sadanandan, Vikas Kumar Anand, Arunan Murali, Ganapathy Krishnamurthi

**Affiliations:** 1 Department of Radiology and Imaging Sciences, Sri Ramachandra Institute of Higher Education and Research, Porur, Chennai, 600 116 India; 2 Department of Engineering Design, Indian Institute of Technology-Madras, Chennai, 600 036 India

**Keywords:** Health care, Medical research, Oncology

## Abstract

In an observational study conducted from 2016 to 2021, we assessed the utility of radiomics in differentiating between benign and malignant lung nodules detected on computed tomography (CT) scans. Patients in whom a final diagnosis regarding the lung nodules was available according to histopathology and/or 2017 Fleischner Society guidelines were included. The radiomics workflow included lesion segmentation, region of interest (ROI) definition, pre-processing, and feature extraction. Employing random forest feature selection, we identified ten important radiomic features for distinguishing between benign and malignant nodules. Among the classifiers tested, the Decision Tree model demonstrated superior performance, achieving 79% accuracy, 75% sensitivity, 85% specificity, 82% precision, and 90% F1 score. The implementation of the XGBoost algorithm further enhanced these results, yielding 89% accuracy, 89% sensitivity, 89% precision, and an F1 score of 89%, alongside a specificity of 85%. Our findings highlight tumor texture as the primary predictor of malignancy, emphasizing the importance of texture-based features in computational oncology. Thus, our study establishes radiomics as a powerful, non-invasive adjunct to CT scans in the differentiation of lung nodules, with significant implications for clinical decision-making, especially for indeterminate nodules, and the enhancement of diagnostic and predictive accuracy in this clinical context.

## Introduction

The differentiation between benign and malignant pulmonary nodules is critical to oncological diagnosis and treatment planning. Traditional methods rely on histopathological confirmation or guidelines such as the Fleischner Society Guidelines, 2017. However, these approaches can be invasive and time-consuming. Radiomics is an emerging field focusing on extracting measurable characteristics from medical images and transforming them into analyzable data. It has exciting potential for a better understanding of tumor characterization and behavior. Unlike traditional visualization of imaging features, radiomics can extract a significantly larger number of imaging features with higher precision and consistency^1^. This field is emerging as a promising technique for lesion characterization. This study aims to explore the utility of radiomics in differentiating between benign and malignant pulmonary nodules, employing machine learning algorithms to enhance diagnostic accuracy.

By definition, a lung nodule is a rounded or irregular opacity, which may be well or poorly defined, measuring ≤ 3 cm in diameter, surrounded by aerated lung on radiological imaging^2^. The etiology of pulmonary nodules is varied and includes benign non-neoplastic causes like infections (granulomas, round pneumonia, septic emboli), benign noninfectious causes (amyloidoma, subpleural lymph nodules, rheumatoid nodules, Wegner’s granulomatosis), benign tumors (hamartoma, carcinoid, neurofibroma, etc.) and malignant neoplasms (primary lung carcinoma, lymphoma, and metastasis)^3^. It is extremely important to distinguish between benign and malignant pulmonary nodules as patient management and clinical outcomes are dependent on this. Early and accurate diagnosis plays an important role in the treatment of cancer. Malignant lung nodules are related to higher mortality and decreased survival rates. Therefore, to distinguish between benign and malignant nodules, accuracy and reproducibility of diagnosis are essential^4^.

Detecting indeterminate pulmonary nodules, often discovered incidentally or during computed tomography (CT)-based lung screening, poses significant difficulties in diagnosis and management. The traditional assessment of nodules relies on discernible visual indicators such as size, borders, shape, and location. The imaging features of benign and malignant lung nodules have considerable overlap, and to achieve better distinction, several additional imaging techniques such as dynamic contrast material-enhanced CT, positron emission tomography (PET), and single-photon emission computed tomography (SPECT) with radioactive tracers have been employed^5^. Currently, image-guided biopsies, though an invasive approach, stands as a problem-solving tool for indeterminate pulmonary nodules. However, unlike biopsies, radiomics feature extraction is non-invasive and it provides three-dimensional information regarding the nodule^6,7^. Our study focuses on examining the radiomic features in benign and malignant pulmonary nodules, and their role in distinguishing between them. We have aimed in our study to utilize radiomic-based feature extraction on images, followed by machine learning-based classification to study the role of radiomics/machine as a non-invasive adjunctive to CT in the evaluation of pulmonary nodules and assess its utility in differentiating between benign and malignant pulmonary nodules. This distinction is of particular importance in guiding toward appropriate patient management and treatment.

## Materials and methods

### Data acquisition

#### Data collection

A cross-sectional observational study (retrospective and prospective) was performed in our department after obtaining prior approval from the Institutional Research Ethics Committee—Sri Ramachandra Institute of Higher Education and Research (CSP–MED/19/SEP/56/122) and all methods were performed in accordance with relevant guidelines and regulations.

**Figure 1:**
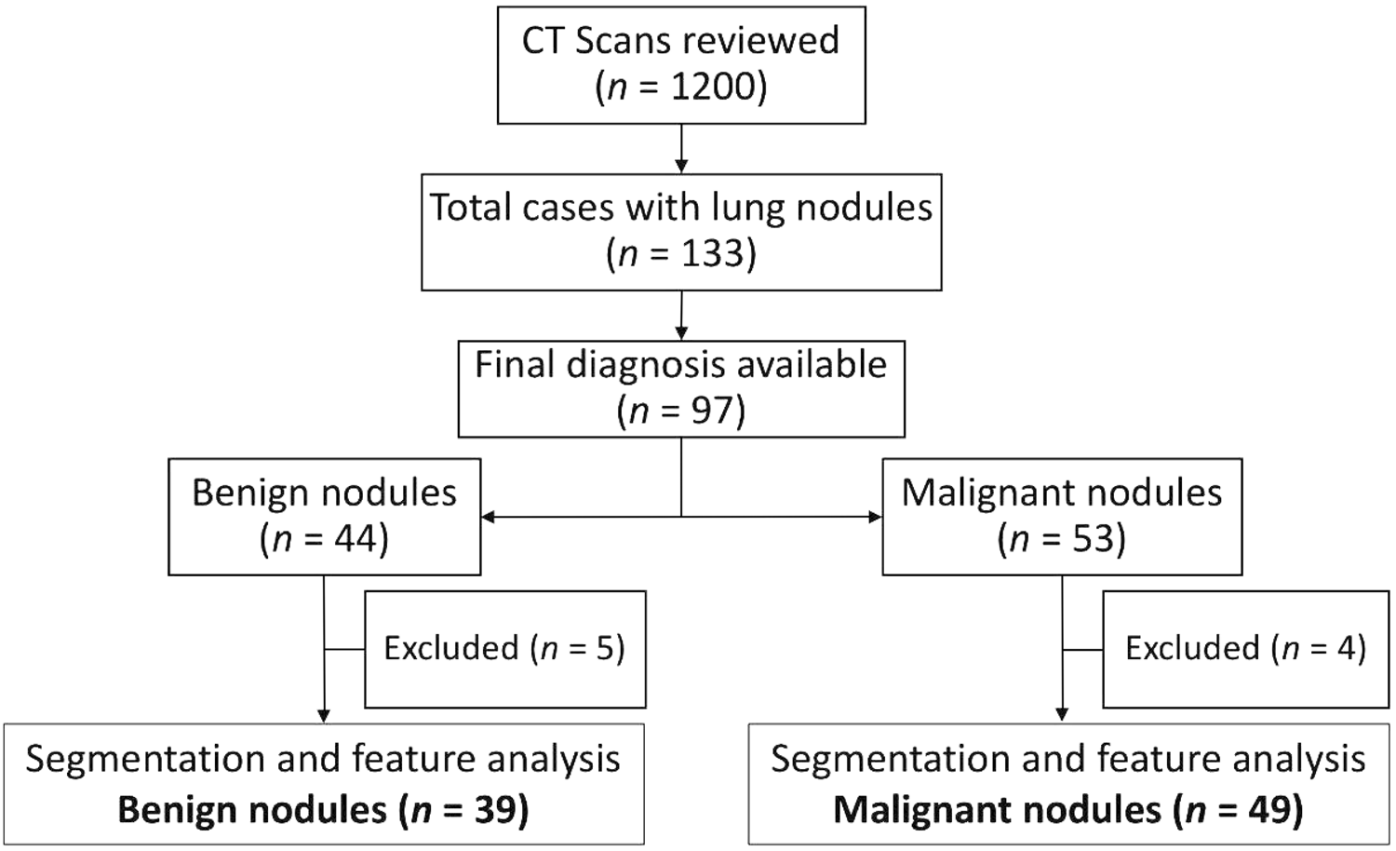
Flow diagram of patient selection.

We initially reviewed CT thorax scans of 1200 patients as shown in Fig. 1. Solid Pulmonary nodules (3–30 mm) were present in 133 cases. The final diagnosis was available in 97 patients, of whom 44 were benign and 53 were malignant pulmonary nodules. The final diagnosis was available in all of these patients. The benign lesions were diagnosed based on histopathological diagnosis or correlation with clinical features and follow-up as per the Fleisher’s Society guidelines^8^. The final diagnosis in primary malignant lesions was arrived at based on histopathological diagnosis, and metastasis was based on the histopathological diagnosis of the lung nodule or primary tumor. However, 4 cases with malignant nodules and 5 cases with benign nodules were excluded because they were infiltrating the ribs and/or had calcifications within. Subsequently, the Digital Imaging and Communications in Medicine (DICOM) images of 39 benign nodules and 49 malignant nodules were subjected to segmentation analysis and radiomics post-processing, and feature extraction. Only patients in whom no other synchronous lung pathology was identified were included in order to prevent overlap of pathologies. Patients with subsolid pulmonary nodules and Nodules with calcification were excluded from the study in order to compare purely solid pulmonary nodules.

#### CT acquisition

High-resolution computed tomography (HRCT) examinations were performed using one of the following multi-slice computed tomography (MDCT) scanners: GE EVO evolution 128 slices (GE Healthcare, Princeton); Phillips-brilliance 16 (Philips Medical Systems, Cleveland); and PET/CT scanner: Siemens biograph horizon (Siemens AG, Munich). HRCT images were obtained during breath-hold using the following parameters: 200 mA and 120 kV. The reconstruction intervals and section thickness were 0.65–0.80 mm. The window width was 1500 and the window level was − 700. Subsequently, the CT images were sent to a picture archiving and communication system (PACS) to be interpreted by radiologists at workstations. However, the radiomic features we are using might depend on the variation in acquisition and reconstruction parameters^9^.

#### Segmentation

**Figure 2:**
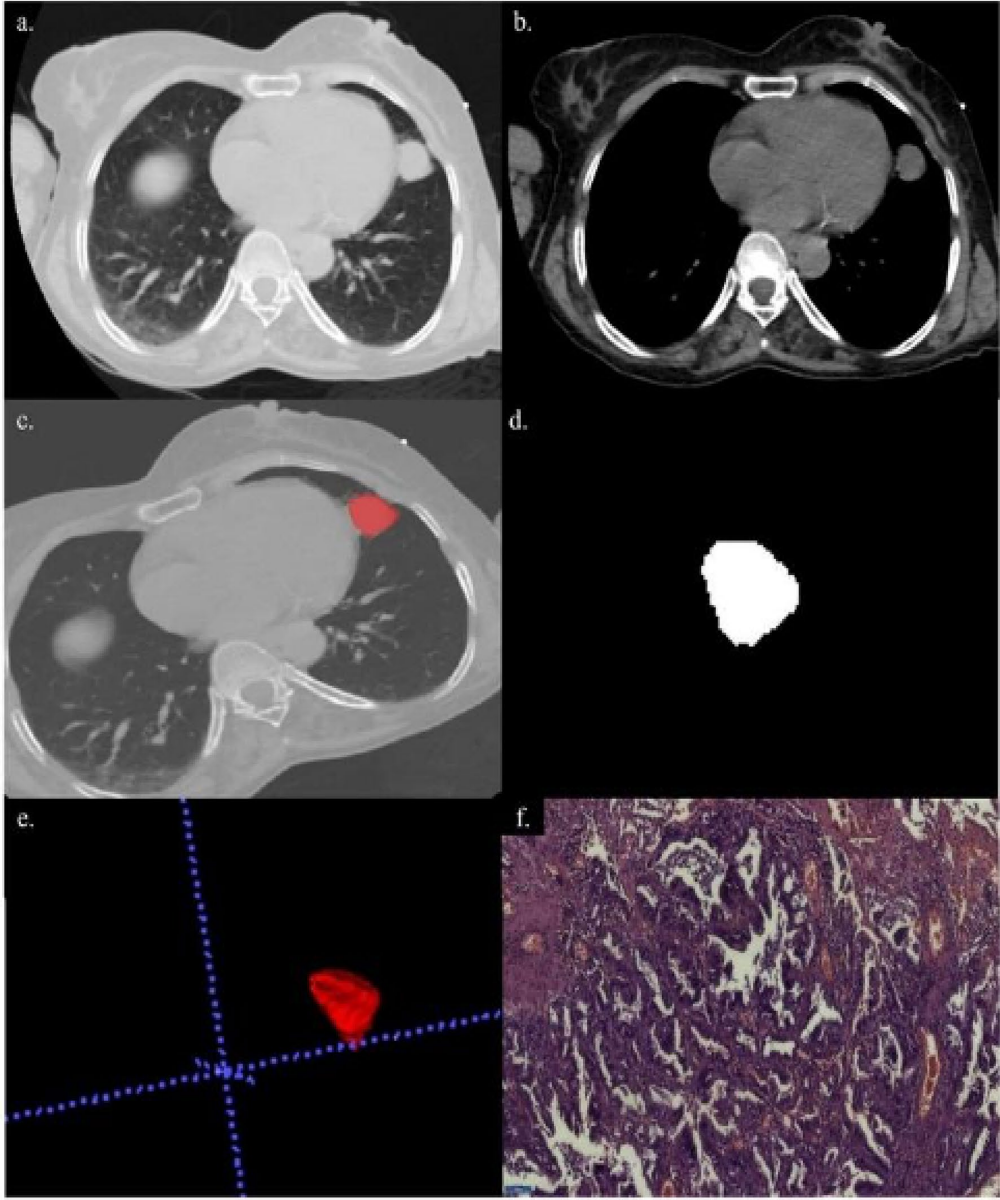
A 54-year-old female with complaints of cough. (**a**, **b**) Axial section of CT thorax in lung window and soft tissue window showing a nodule in the lingular segment of the upper lobe. (**c**) Creation of ROI for segmentation. (**d**) 2D-segmented nodule. (**e**) 3D-volumetric rendering of the nodule. (**f**) HPE: Adenocarcinoma lung.

**Figure 3:**
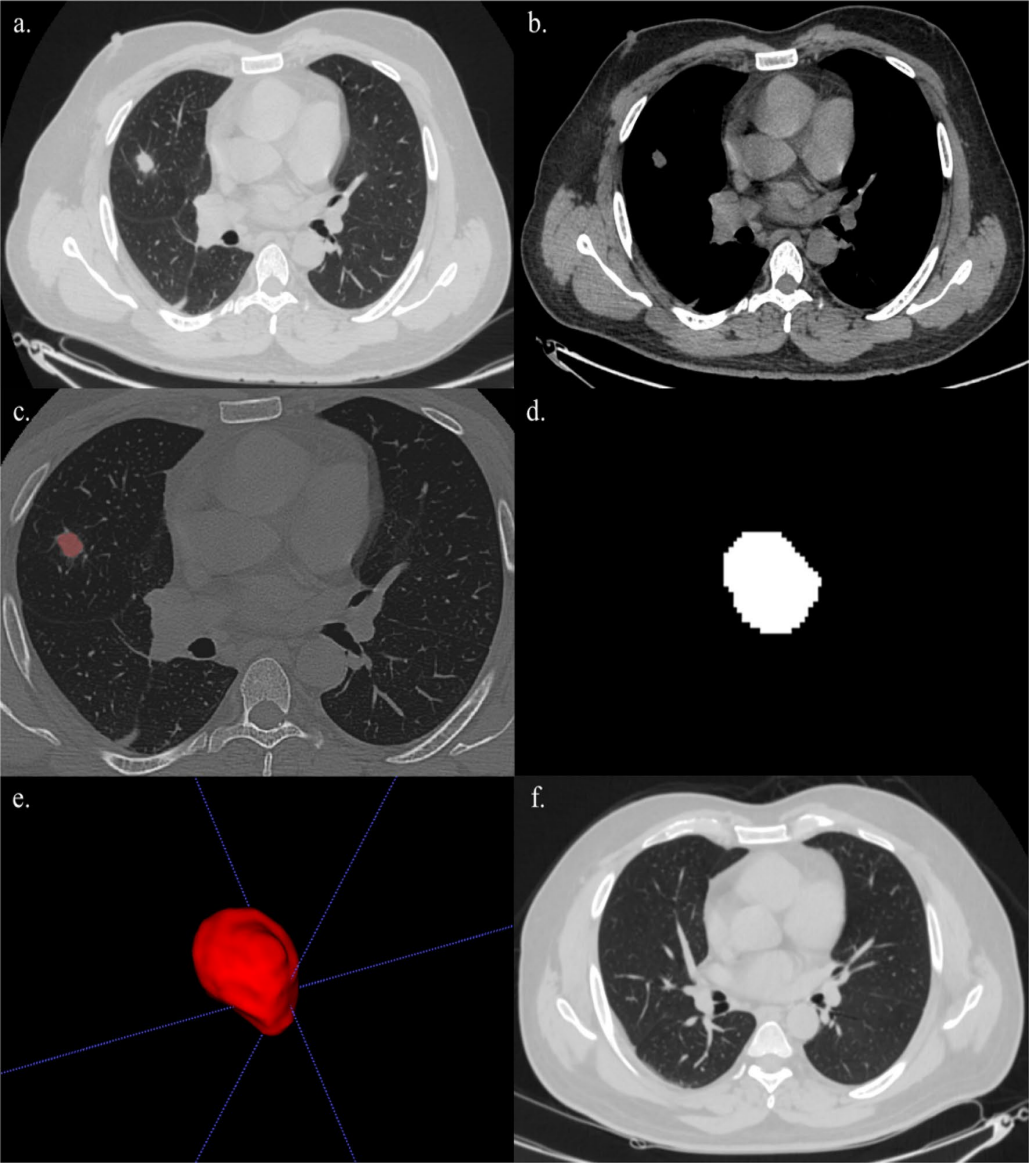
A 53 year-old male with complains of cough and fever. (**a**, **b**) Axial section of CT thorax in lung window and soft tissue window showing nodule in the latreral segment of right middle lobe. (**c**) Creation of ROI for segmentation. (**d**) 2D segmented nodule. (**e**) 3D volumetric rendering of the nodule. (**f**) Patient was put on a course of antibiotic therapy and follow-up imaging done after 6 months revealed complete resolution of the nodule.

**Figure 4:**
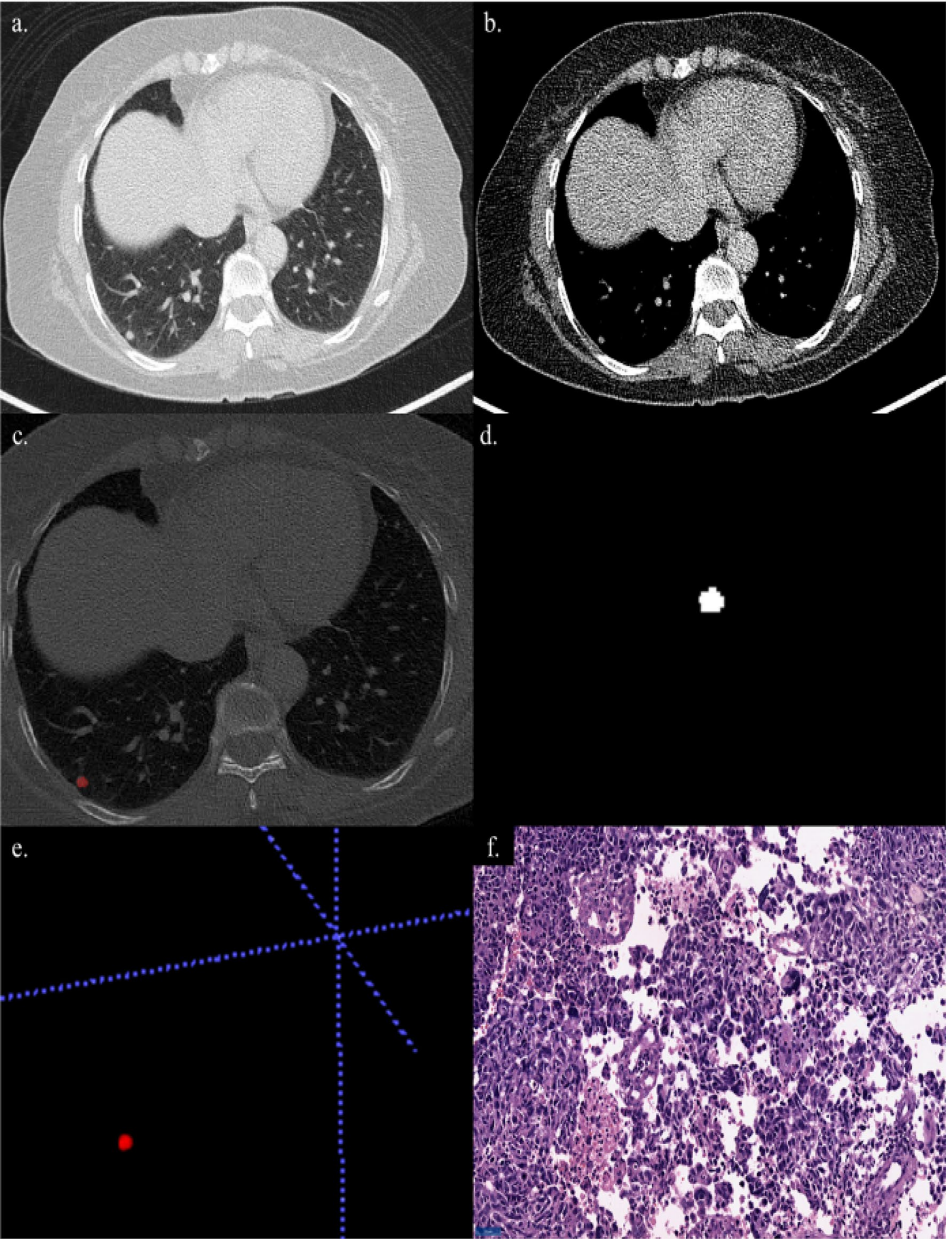
A 66-year-old female with a lump in the right breast. (**a**, **b**) Axial sections of CT thorax showing a nodule in the posterio-basal segment of the right lower lobe. (**c**) Creation of ROI for segmentation. (**d**) 2D-segmented nodule. (**e**) 3D-volumetric rendering of the nodule. (**f**) A final diagnosis—metastasis HPE: Primary malignancy—carcinoma breast.

Segmentation of the DICOM images of the pulmonary nodules was done by a radiologist using Insight Segmentation and Registration Toolkit (ITK-SNAP) software^10^_._ and was verified by three radiologists independently. The steps described above are shown in Figs. 2, 3, 4.

### Demographic details

#### Age distribution

**Figure 5:**
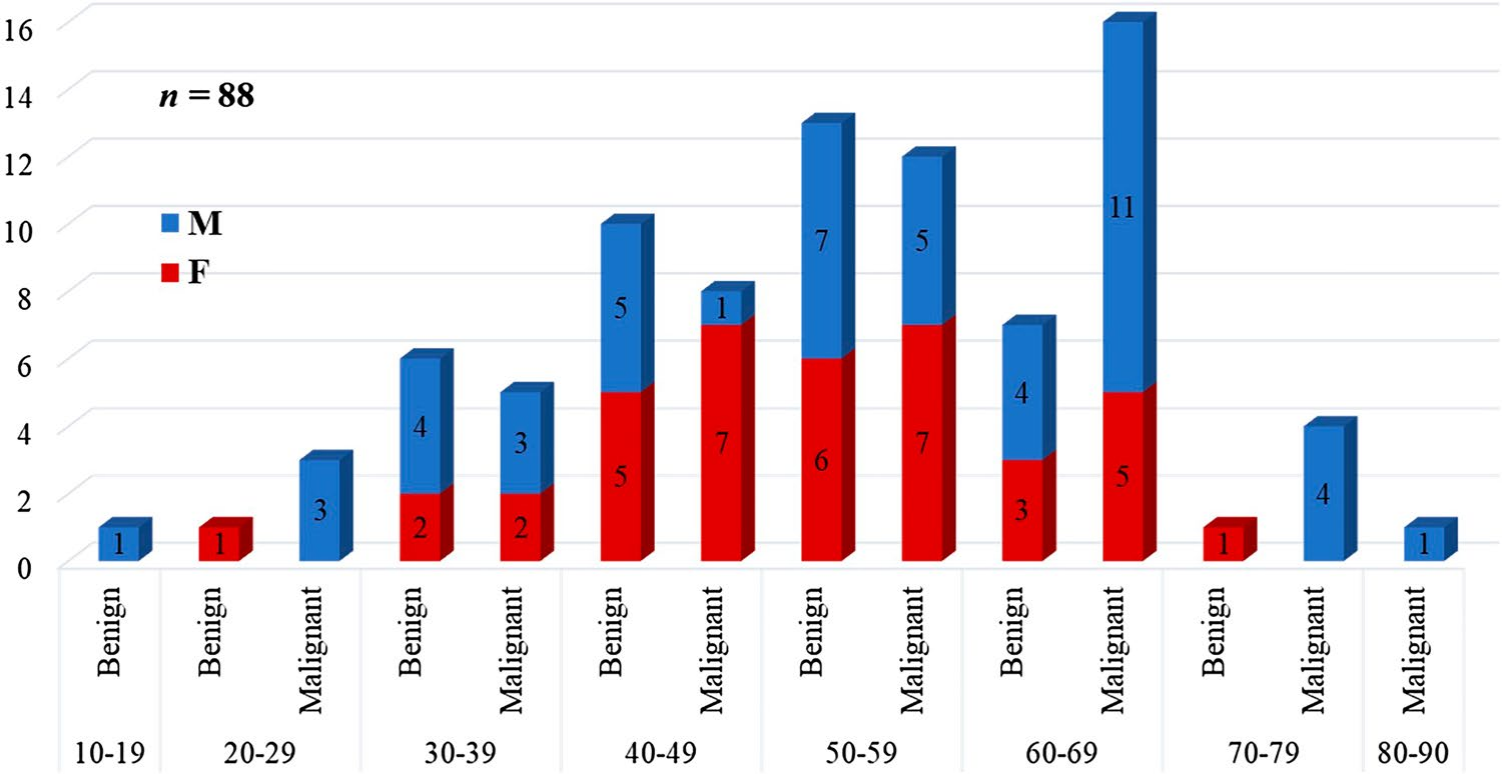
Age–gender distribution in benign and malignant pulmonary lesions.

The age of the study group ranged from 18 to 83 years. The peak incidence of the pulmonary nodule was noted in the sixth decade. Our data comprised 49 males and 39 females. The age and gender distribution of the patients included in our study are illustrated in Fig. 5.

### Methods and training algorithms

#### Data pre-processing and radiomic feature extraction

The original data in our study was in the DICOM format, which is a standard format for storing medical imaging data. However, the Neuroimaging Informatics Technology Initiative (NIfTI) format has been used often by researchers in the medical imaging community due to its flexibility and ease of use. Moreover, the NIfTI format is widely utilized in the computational medical imaging community because it enables direct conversion to various desirable formats. Therefore, an important step in preprocessing the data was to convert the DICOM image we had to the NIfTI format. This conversion allowed us to utilize the numerous existing algorithms developed for the NIfTI format, facilitating further processing of the images. Additionally, the converted images were saved in compressed gzipped files (.nii.gz) to reduce storage space requirements. The masks obtained for our study were already in the NIfTI format.

**Figure 6:**
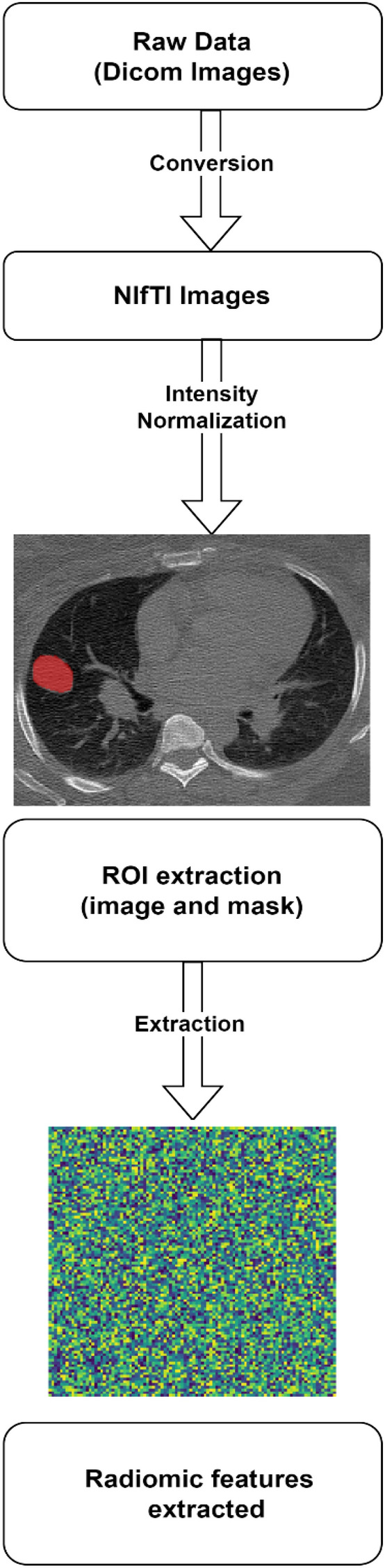
The data pre-processing steps employed to extract radiomic features.

Segmented lung nodules along with the NIfTI images were used to extract different types of radiomic features as shown in Fig. 6. Radiomics uses data characterization algorithms to extract a variety of features from medical images. These features have the potential to reveal tumor patterns and features not apparent to the naked eye. For our analysis, we utilized 14 shape-based features, 18 first-order features, and 69 texture-based features, resulting in a comprehensive set of 101 features. Texture features have been found to be one of the most important imaging features in the field of radiomics^11^. Texture-based features were of three types namely, grey-level co-occurrence matrix (GLCM) features (27 features), grey-level run-length matrix (GLRLM) features (16), and grey-level size zone matrix (GLSZM) features (16)^12,13^. Each radiomics feature was given a feature rank based on a random forest classifier. Out of 101, the top 10 features were selected by the random forest classifier used for classification algorithms^14^.

### Classification algorithms

#### Machine learning algorithms

In our study, we employed various machine learning-based classification algorithms, including k-Nearest Neighbors, support vector machine (SVM)^15^, decision trees, multi-layer perceptron (MLP), and Naive Bayes, to analyze our data. We utilized both linear and radial basis function kernels for SVM, as well as linear discriminant analysis (LDA) and quadratic discriminant analysis (QDA). In addition, we used MLP classifiers with different activation functions, such as rectified linear unit (RELU), identity, and hyperbolic tangent (tanh). Furthermore, we compared the performance of these classifiers with a simple two-hidden-layer neural network.

#### Data splitting and cross-validation

The dataset was randomly partitioned into a training set and a test set, maintaining an 80:20 ratio. The classifiers were trained on the training sets, and their performance was evaluated on unseen test sets. A rigorous five-fold cross-validation was performed to ensure the robustness of the testing.

#### Performance metrics

The classifiers' performance was assessed using confusion matrices, from which the following metrics were derived:

- *Accuracy* The proportion of true results among the total number of cases examined.
- *Sensitivity* The ability of the model to identify true positives.
- *Specificity* The ability of the model to identify true negatives.
- *Precision* The proportion of true positives among the total predicted positives.
- *F1-Score* A measure of a model's accuracy that considers both precision and recall.
- *ROC-AUC* The Receiver Operating Characteristic (ROC) Area Under the Curve (AUC) measures a classifier's capacity to differentiate between positive and negative classes, independent of any specific threshold.

#### Ensemble modeling with XGBoost

Upon observing superior performance from tree-based models, the data was further trained on an ensemble model, specifically Extreme Gradient Boosting (XGBoost). This advanced approach was implemented to elevate the model's performance, and its efficacy was assessed using the aforementioned metrics.

#### Deep learning algorithms

We employed advanced Convolutional Neural Networks (CNNs) such as ResNet50, DenseNet, and Vision Transformer for 3D pulmonary nodule classification, focusing on the Region of Interest (ROI). These models were pre-trained on the MedMNIST dataset and then fine-tuned to classify pulmonary nodules.

### Ethical clearance

The study was performed after obtaining prior approval from the Institutional Research Ethics Committee—Sri Ramachandra Institute of Higher Education and Research (CSP–MED/19/SEP/56/122) and all methods were performed in accordance with relevant guidelines and regulations.

### Informed consent

Informed consent was obtained from all subjects and / or their legal guardians involved in the study.

## Results

### Case distribution

**Figure 7:**
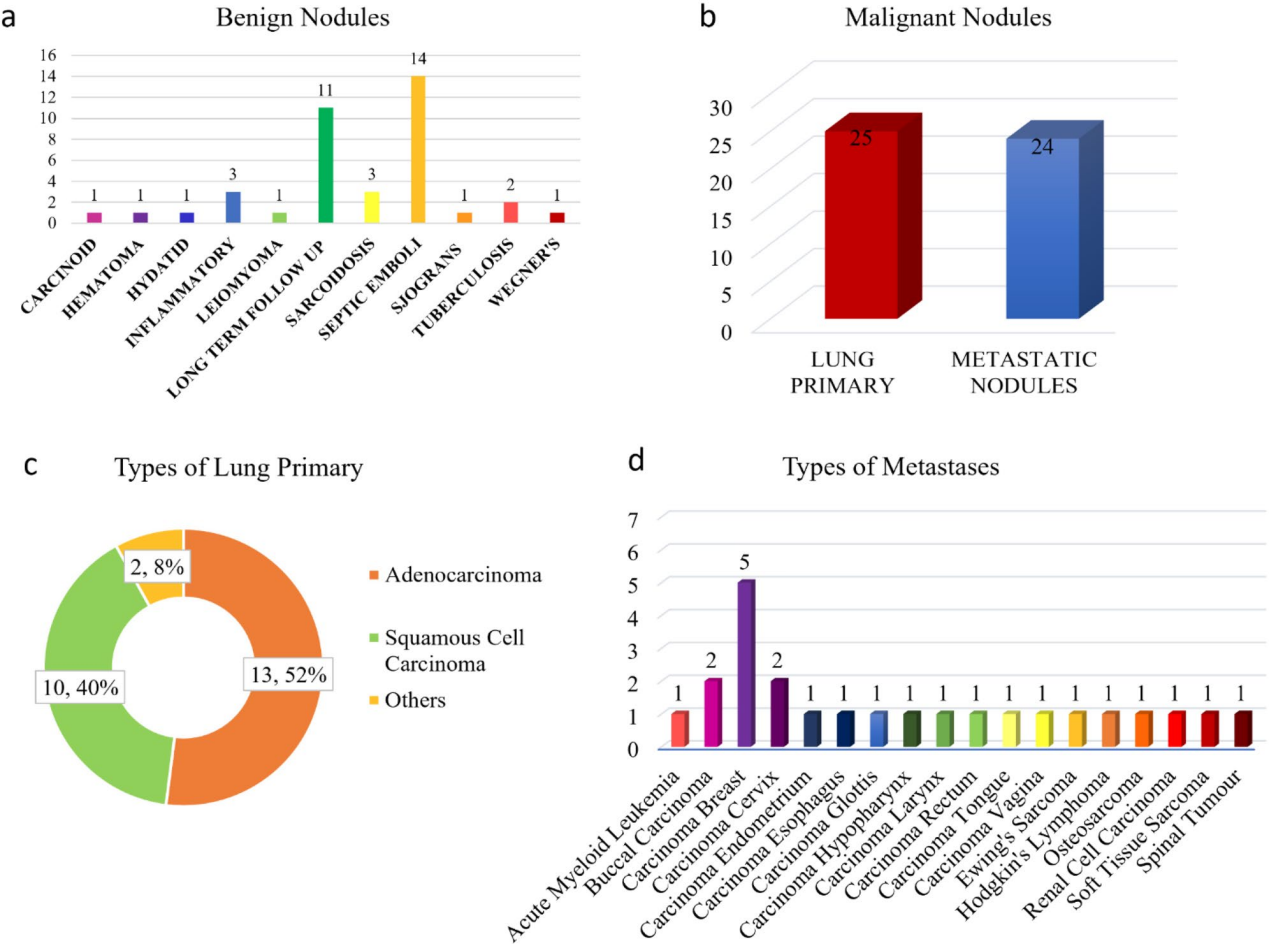
Case distribution of benign and malignant nodules.

A 44% of the nodules were benign pulmonary nodules and the remaining were malignant pulmonary nodules. 51% of the malignancies were primary lung malignancies whereas the rest were metastasis. Among the various metastases, the majority were from breast carcinoma, and the remaining were from various other primary organs. The distribution of the diverse range of cases is depicted in Fig. 7. Of the benign lesions (n = 39) which underwent radiomic analysis, 14 cases were of septic emboli, 11 cases of benign lesions followed up on a long-term basis as per Fleishner society guidelines, 3 each of sarcoidosis and inflammatory etiology, 2 cases of pulmonary tuberculosis followed by 1 case each of benign carcinoid, hematoma, hydatid disease, Sjogren’s syndrome, and Wegner’s granulomatosis.

The malignant cases (*n* = 49) included 25 cases of primary lung malignancies and 24 cases of metastasis. The primary lung malignancies included adenocarcinoma (*n* = 13), squamous cell carcinoma (*n* = 10), and one case each of malignant carcinoid tumor and small cell carcinoma respectively. Among the various metastasis, 5 were from carcinoma breast, 2 each from buccal carcinoma, carcinoma cervix, and Hodgkin’s lymphoma, and one case each of acute myeloid leukemia, carcinoma endometrium, carcinoma esophagus, carcinoma glottis, carcinoma hypopharynx, carcinoma larynx, carcinoma rectum, carcinoma tongue, carcinoma vagina, Ewing’s sarcoma, osteosarcoma, renal cell carcinoma, soft tissue sarcoma, and vertebral lymphoma.

### Classification results

#### Machine learning algorithms

**Figure 8:**
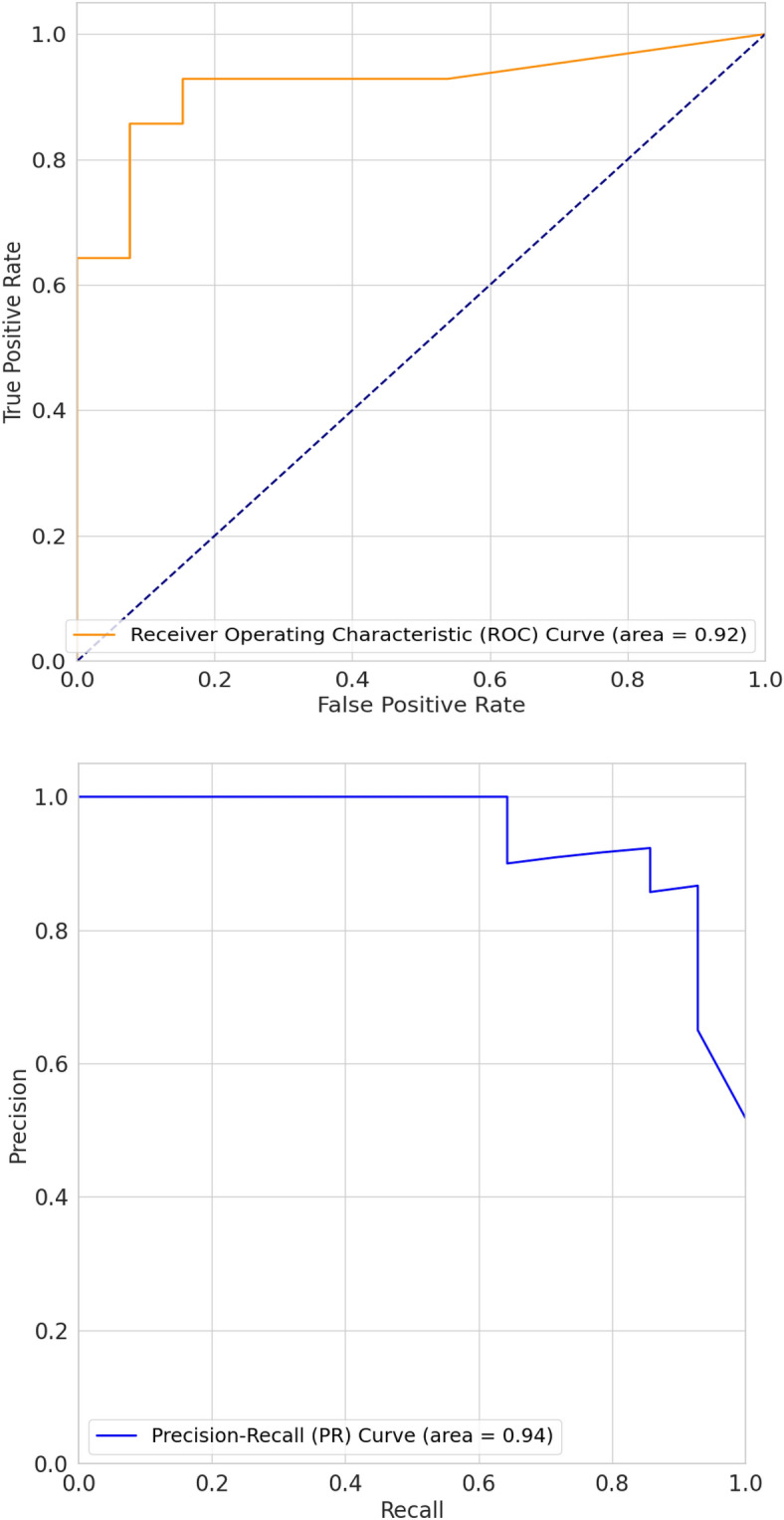
Receiver operating characteristic ROC and PR curves.

The performance of the different classifiers was calculated from the test data, using the radiomic features that were extracted in tabular format. As observed from Table 1, the Decision Tree model outperformed the other classifiers on the test data. The tree-based models exhibited superior performance for our data, prompting further exploration with Extreme Gradient Boosting (XGBoost). In our analysis, the optimal threshold determined from the ROC curve was 0.5752 for computing the performance metrics. This advanced ensemble technique yielded remarkable results, achieving an accuracy of 89% and AUC of 88.7%, along with sensitivity, precision, and F1-Score all registering at 89% and specificity of 84.9% for the dataset. The Receiver Operating Characteristic (ROC) curve and the Precision-Recall (PR) curve are depicted in Fig. 8. The Area Under the ROC Curve (ROC-AUC) for our classifier is 0.92. However, the Area Under the Precision-Recall Curve (PR-AUC) is 0.94, indicating good precision and recall performance by the classifier. This outcome represents a significant enhancement compared to a previous similar study by Tu et al.^16^, which reported an accuracy of 79% and AUC of 80% with corresponding sensitivity, specificity, precision, and F1-Score values of 88%, 64%, 80%, and 83%, respectively. We benchmarked our results against the accuracies reported in previous deep learning studies, as outlined in the paper by Tomassini, Selene et al. and Riquelme and Akhloufi^17,18^. Notably, the highest accuracy achieved in these studies was 94% for a nodule volume of 32 × 32 × 6 on the LIDC-IDRI dataset using a Multi-scale multi-task 3D CNN^17^. They have also reported different accuracies of 84%, 87%, 90%, and 93% for the same dataset but with different nodule volumes^17,18^. In our study, using radiomic features extracted from nodule volumes (full nodule), we attained an accuracy of 88%. This outcome is notable, particularly given the constraints of our smaller dataset, which inherently presents challenges for robust feature extraction using 3D CNNs. Additionally, it's worth emphasizing that all the nodules in our study were histopathologically confirmed. The utilization of XGBoost with radiomic features in our study underscores its potential as a robust tool for differentiating benign and malignant pulmonary nodules especially when the dataset is small. Moreover, Monte Carlo repetitions were performed for the XGBoost classifier, and the outcomes are presented in Table 2.

**Table 1:** Different performance metrics for diverse classifiers were obtained on the test dataset.

Classifiers	Accuracy	Sensitivity	Specificity	Precision	F1 score
KNN	0.684	0.667	0.7147	0.8	0.7277
Linear SVM	0.523	0.667	0.286	0.6156	0.64
RBF SVM	0.7	0.769	0.571	0.769	0.769
Decision tree	0.789	0.75	0.857	0.9	0.818
Random forest	0.714	0.692	0.75	0.818	0.75
Adaboost	0.684	0.666	0.714	0.8	0.727
Naive Bayes	0.55	0.333	0.875	0.8	0.470
LDA	0.684	0.583	0.857	0.875	0.70
QDA	0.6	0.416	0.875	0.833	0.555
MLP classifier ReLU	0.632	0.583	0.714	0.778	0.667
MLP classifier Identity	0.7	0.615	0.857	0.889	0.727
MLP classifier Tanh	0.65	0.615	0.714	0.8	0.695
Neural network	0.78	0.75	0.75	0.74	0.725
XGBoost	0.89	0.89	0.849	0.89	0.89

**Table 2:** Results from Monte Carlo repetitions for the XGBoost classifier.

Performance metrics	Mean	Standard deviation
Accuracy	0.842	0.029
Sensitivity	0.846	0.029
Specificity	0.878	0.031
Precision	0.852	0.023
F1 score	0.846	0.025

#### Deep learning classifiers

As indicated in Table 3, our deep learning models exhibited lower performance compared to the radiomic-based machine learning models. This suboptimal performance could be attributed to the limited amount of data available for training. Lacking sufficient data to learn appropriate feature extraction, the deep learning models consequently performed poorly on the unseen dataset. Indeed, conventional radiomic analyses possess inherent limitations. It is, however, crucial to highlight that our dataset is relatively small. We have explored various transfer learning methodologies, employing architectures such as ResNet50, DenseNet, and Vision Transformer 3D. Features were extracted from these pre-trained models to facilitate classification, and the outcomes are delineated in Table 3. Given the constraints of our dataset's size, fine-tuning was challenging. Consequently, the features extracted from the deep learning models were not particularly adept at distinguishing between benign and malignant nodules. This underscores our observation: in scenarios with limited datasets, traditional radiomic features tend to outperform those derived from deep learning models.

**Table 3:** Different performance metrics obtained from the deep learning-based models.

Classifiers	Accuracy	Sensitivity	Specificity	Precision	F1 score
DenseNet	0.624	0.567	0.414	0.524	0.5942
ResNet50	0.453	0.473	0.682	0.361	0.409
Vision transformer	0.48	0.38	0.66	0.52	0.4249

### Interpretability

**Figure 9:**
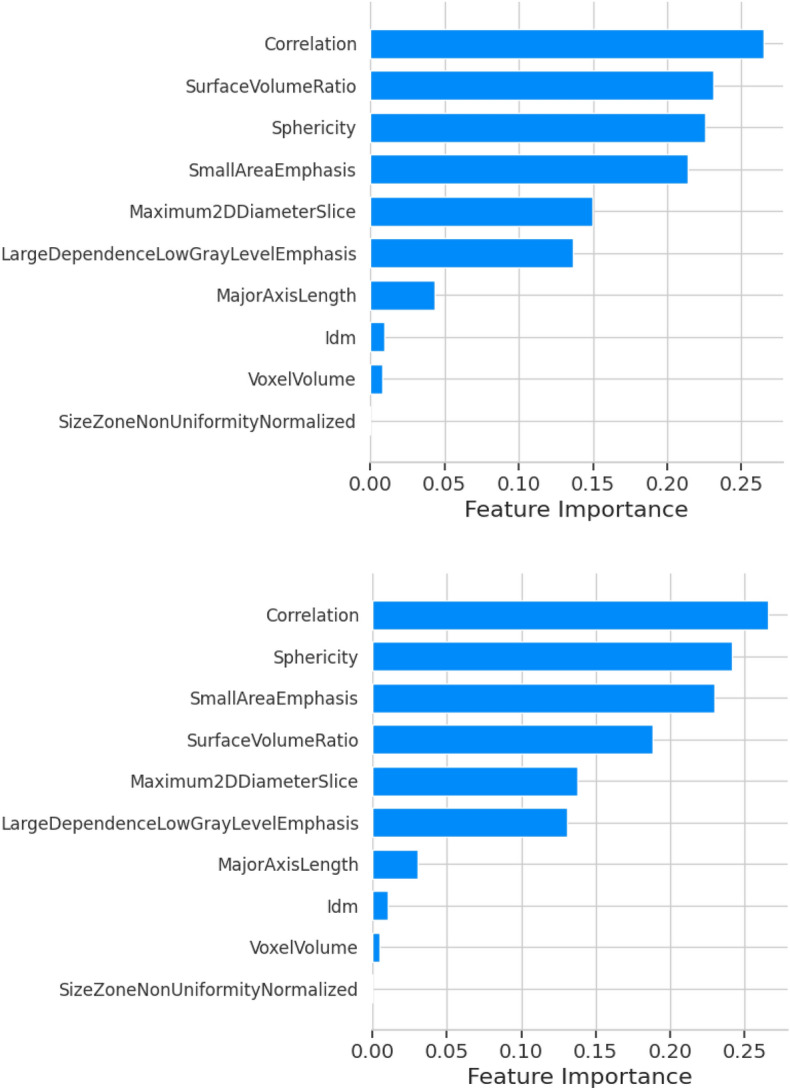
Importance of different radiomic features using SHAP (top—train; bottom—test).

In the intricate task of classifying benign and malignant lung tumors, radiologists traditionally rely on visual cues and morphological characteristics observed in CT scans. Our study has identified key radiomic features that align with these clinical observations, enhancing the interpretability of the machine learning model. Utilizing SHAP (SHapley Additive exPlanations) in Python, we could quantify the top five feature's contribution to the model's prediction as shown in Fig. 9^19^. The figure illustrates the important features derived from the Shapley values for both the training and test datasets. In both cases, 'Correlation' emerges as the most significant feature. While the majority of features maintain consistent rankings between the train and test datasets, there are a few notable deviations. Specifically, the 'Surface Volume Ratio', which is the second most important feature in the training set, ranks as the fourth most important in the test set. Conversely, the features ranked third and fourth for the training data ascend to the second and third positions respectively in the test data. Beyond these exceptions, the significance rankings of other features remain largely consistent between the two datasets.

- *Correlation*, reflecting linear dependencies of gray-level values, measures the linear relationship of the grayscale intensity level in an image. It aligns with radiologists' understanding of texture and structure, as a high correlation often suggests a more structured and homogenous texture, while a lower correlation might indicate more complexity, typically associated with malignancy^20^.
- *Sphericity* measures how closely a shape resembles a sphere, reflecting the tumor's shape. Benign nodules are often more spherical, while primary lung malignancies might have more irregular shapes. This feature provides an essential connection to the visual observations of radiologists in their evaluations^8,21^.
- *Small area emphasis*, indicative of tumor heterogeneity, measures the distribution of small-size areas in the image. It resonates with radiologists' assessments of texture and structure, as benign nodules may present with more uniform textures, while malignant nodules may exhibit more heterogeneous small areas^20^.
- *Surface volume ratio*, providing geometric insights into the tumor's complexity, gives an insight into the surface irregularity of a nodule. A higher surface volume ratio could be indicative of a more irregular surface, often seen in malignant tumors, mirroring the visual cues that radiologists utilize in their evaluations^8,21^.
- *Maximum 2D diameter slice* provides insights into the tumor's size, referring to the largest diameter within a single 2D slice of the tumor. It aligns with the traditional methods radiologists use to gauge tumor size, as larger diameters might be more indicative of malignant tumors, although this is not always the case^8,21^.

SHAP values, derived from cooperative game theory, offer a consistent and locally accurate attribution for each feature, allowing for a subtle understanding of how these features influence the classification. The alignment of these radiomic features with traditional radiological practices not only validates the model but also bridges the gap between machine learning and clinical decision-making. By translating complex model decisions into clinically relevant terms, this approach fosters trust and facilitates the integration of machine learning into radiological practice, potentially enhancing diagnostic accuracy and patient care.

## Discussion

The present study underscores the significant potential of radiomics as a non-invasive tool in differentiating between benign and malignant pulmonary nodules. Achieving an accuracy of 89% with the Extreme Gradient Boosting (XGBoost) algorithm, our findings demonstrate a substantial advancement in the field. This achievement is particularly noteworthy given that our dataset is small as well as it consists exclusively of histopathologically confirmed samples. A cornerstone of our study is the interpretability of the model, facilitated by the extraction and utilization of key radiomic features. These features, encompassing first-order, shape, and texture characteristics, resonate with traditional radiological practices. Tumor texture, identified as the primary predictive feature for malignancy, highlights the critical role of texture-based features in computational oncology. This alignment with visual cues and morphological characteristics that radiologists rely on in CT scans fosters trust and facilitates the integration of machine learning into clinical practice. Radiomics-based machine learning classification unlike biopsies, is non-invasive, three-dimensional, and provides information regarding the entire lesion. Moreover, the training time and computational resources required for these classifiers including extracting features are much less compared to deep convolutional neural network which takes 3D CT scan as input. It makes this method more deployable even if we have only limited computational resources. This emphasizes our finding that in situations where there is a scarcity of data, conventional radiomic features tend to yield better results than features extracted from deep learning models. Moreover, this approach has the capacity to decrease the number of unnecessary benign biopsies, which may result in improved patient results and increased cost-effectiveness. This approach has advantages over traditional biopsy methods, which may result in post-procedural complications such as pneumothorax, pulmonary hemorrhage, or air embolism^22^. Thus, with more multicentric trials and standardization, radiomics in the near future will have an important role in pulmonary nodule diagnosis and aid in precision oncology.

Our study has certain limitations that need to be acknowledged. Our dataset is relatively small and originates from a single medical center. The smaller dataset could in major part be attributed to the fact that the time period of our study overlapped with the COVID—pandemic which led to a reduction in the number of cases available for analysis during the study period. Moreover, some patients with lung nodules on Chest CT were lost to follow-up, and hence, had to be excluded from the study population as a final diagnosis was unavailable in these cases. On the other hand, the radiomic features can be influenced by the acquisition and reconstruction parameters of CT scans. For this reason, we have meticulously detailed the parameters used in our dataset to ensure clarity and reproducibility. In radiomic feature analysis, there are inherent limitations that need to be considered. One such limitation is the discretization of image intensities into predefined bins or levels during the feature extraction process. While this step is essential for the creation of matrices like the Gray Level Co-occurrence Matrix (GLCM) and the Gray Level Run Length Matrix (GLRLM), it can result in a potential loss of nuanced information. Additionally, the analysis predominantly concentrates on the nodule regions, thereby overlooking possible informative aspects from the surrounding areas. Despite these challenges, it is vital to consider these limitations as opportunities for future research improvement. Widespread multicentre trial studies are required to realize the full potential of radiomics in lung nodules. The preliminary account of this study was presented elsewhere^23^.

## Conclusion

In summary, achieving an accuracy of 89% on a limited dataset underscores the robustness of the approach and paves the way for further research and clinical application. The findings hold promise for enhancing patient care and advancing personalized medicine in the management of pulmonary nodules. It is expected to have a real clinical impact, as imaging is routinely used in clinical practice, providing an opportunity to improve decision support in lung nodule management (especially with indeterminate pulmonary nodules) at a low cost and as a non-invasive approach. This emphasizes our finding that in situations where there is a scarcity of data, conventional radiomic features tend to yield better results than features extracted from deep learning models.

## Data Availability

The datasets generated or analysed during the study are available from the corresponding author upon reasonable request.
